# A novel lncRNA, GASL1, inhibits cell proliferation and restricts E2F1 activity

**DOI:** 10.18632/oncotarget.15864

**Published:** 2017-03-03

**Authors:** Lital Gasri-Plotnitsky, Aviv Ovadia, Katerina Shamalov, Tali Nizri-Megnaji, Shimrit Meir, Irit Zurer, Cyrille J. Cohen, Doron Ginsberg

**Affiliations:** ^1^ The Mina and Everard Goodman Faculty of Life Science, Bar Ilan University, Ramat Gan 52900, Israel

**Keywords:** lncRNA, cell cycle, cell proliferation, E2F

## Abstract

The human genome encodes thousands of unique long non-coding RNAs (lncRNAs), many of which are emerging as critical regulators of cell fate. However, their functions as well as their transcriptional regulation are only partially understood. The E2F1 transcription factor induces both proliferation and apoptosis, and is a critical downstream target of the tumor suppressor, RB. Here, we provide evidence that a novel lncRNA named GASL1 is transcriptionally regulated by E2F1; GASL1 levels are elevated upon activation of exogenous E2F1 or endogenous E2Fs. Inhibition of GASL1 expression induced cell cycle progression, and in particular, G1 exit. Moreover, GASL1 silencing enhanced cell proliferation, while, conversely, its ectopic expression inhibited proliferation. Knockdown of GASL1 also enhanced E2F1-induced apoptosis, suggesting the existence of an E2F/GASL1 negative feedback loop. In agreement with this notion, silencing of GASL1 led to increased levels of phosphorylated pRB and loss of Rb impaired the effect of GASL1 silencing on G1 exit. Importantly, xenograft experiments demonstrated that GASL1 deletion enhances tumor growth. Moreover, low levels of GASL1 are associated with decreased survival of liver cancer patients. Taken together, our data identify GASL1 as a novel lncRNA regulator of cell cycle progression and cell proliferation with a potential role in cancer.

## INTRODUCTION

The human genome expresses tens of thousands of long non-coding RNAs (lncRNAs), which are >200 bases in length but lack significant open reading frames [1]. In general, lncRNAs are poorly conserved across species compared to protein coding genes; however, thousands of lncRNAs are evolutionary conserved [2]. Expression of lncRNAs is often restricted to specific cell types [1] and many lncRNAs function in cellular differentiation and development [3] while others play a critical role in regulation of cell cycle progression and apoptosis [4]. In particular, a number of lncRNAs were shown to function in cell cycle progression via regulation of critical cell cycle regulators such as the cyclins, CDKs, CDK inhibitors, pRB, and p53 [5–8]. Moreover, many lncRNAs are frequently aberrantly expressed in various human cancers, with potential roles in both oncogenic and tumor suppressive pathways [9–14]. It is now considered likely that ncRNAs of this class are a significant feature of normal cellular networks.

The promoters of many lncRNAs are bound and regulated by transcription factors known to influence mRNA transcription, including cancer-related transcription factors, such as p53 [15, 16], Myc [17, 18] and E2F [19–22].

Here, we identified a novel lncRNA, which we named GASL1 (Growth-arrest Associated lncRNA 1). We show that expression of GASL1 is upregulated by both ectopically expressed, as well as endogenous E2F, most probably via an indirect mechanism. Silencing of GASL1 enhances G1/S transition and cell proliferation, while its ectopic expression inhibits cell growth. Silencing of GASL1 also enhanced E2F1-induced apoptosis, suggesting the existence of an E2F/GASL1 negative feedback loop. In agreement with this notion, silencing of GASL1 resulted in reduced levels of the CDK inhibitor, p21, and elevated pRB phosphorylation. Moreover, loss of Rb impaired the effect of GASL1 silencing on G1 exit. Our data also show that GASL1 deletion enhances xenograft growth and, importantly, low levels of GASL1 are associated with decreased survival of liver cancer patients.

## RESULTS

**Figure 1:**
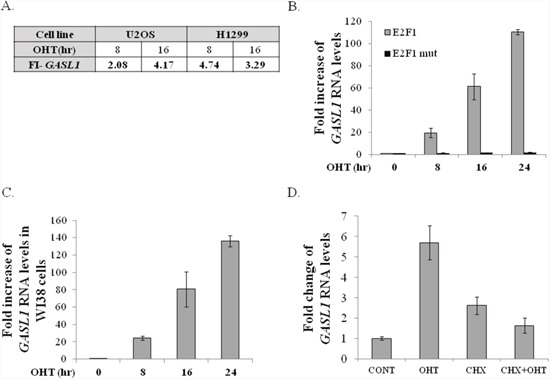
Ectopic E2F1 upregulates GASL1 RNA levels **(A)** U2OS and H1299 cells containing conditionally active E2F1 were induced to activate E2F1 by addition of 4-hydroxytamoxifen (OHT) for the times indicated. RNA was extracted and RNA deep sequencing analysis was employed. FI- fold increase in *Gasl1* RNA levels after E2F1 induction, as determined by RNA sequencing. **(B)** U2OS cells expressing either ER-E2F1 (ER-E2F1) or mutant ER-E2F1-E132 were left untreated or incubated with 4-OHT (100 nM) for the indicated times. RNA was extracted, and *Gasl1* RNA levels determined by Real-time RT-PCR, and normalized to *Gapdh* levels. **(C)** WI38 cells expressing ER- E2F1 were left untreated or incubated with 4-OHT (100 nM) for the indicated times. RNA was extracted, and *Gasl1* RNA levels determined by Real-time RT-PCR, and normalized to *Gapdh* levels. **(D)** U2OS cells stably expressing ER-E2F1 were treated with OHT (100nM) for 8hr (OHT) or left untreated, in the presence or absence of 10 μg/ml cycloheximide (CHX). RNA was extracted, and the level of *Gasl1* RNA determined by real time qPCR and normalized to *Gapdh* levels.

GASL1 (partially overlapping ENST00000517910) is a 3435 bases long intergenic lncRNA, whose mature RNA is 1536 bases long, that is located on chromosome 8 (chr8:103,819,901-103,823,335) at a position corresponding to band q22.3 on a somatic map, and is transcribed from the plus strand and consists of 4 exons (Supplementary Figure 1). GASL1 was first identified as an E2F1-regulated lncRNA in an RNA Seq.-based screen performed using the human osteosarcoma cell line U2OS and the human lung carcinoma cell line H1299, which express conditionally active E2F1, namely ER-E2F1 [20]. RNA-Seq. data demonstrated that upon activation of ectopic E2F1 RNA levels of GASL1 were increased about 4 fold in both cell lines (Figure 1A). Next, we validated the expression data obtained from the RNA-sequencing by performing Real-Time PCR, using RNA of cells expressing the conditionally active E2F1. The real-time PCR analysis demonstrated that activation of the ectopic E2F1 increased expression of GASL1 in both U2OS and H1299 cell lines, as well as in WI38 human embryonic lung fibroblasts (Figure 1B, 1C and Supplementary Figure 2). Activation of mutated E2F1, which does not bind DNA, did not significantly affect GASL1 expression, demonstrating that the E2F1-induced upregulation of GASL1 requires E2F1 transcriptional activity (Figure 1B). To further examine whether E2F1 regulates GASL1 directly or indirectly, ectopic E2F1 was activated in the presence of cycloheximide, an inhibitor of protein synthesis. The E2F1-induced increase in GASL1 RNA levels was significantly inhibited by cycloheximide, indicating that GASL1 activation by E2F1 occurs via an indirect mechanism (Figure 1D).

**Figure 2:**
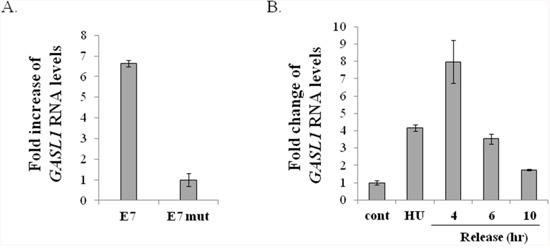
Expression of *GASL1* is regulated by endogenous E2F and during the cell cycle **(A)** WI38 cells were infected with a retrovirus expressing either wild-type E7 (E7) or an RB-binding–deficient E7 mutant (E7 mut). RNA was extracted, and *Gasl1* RNA levels determined by Real-time RT-PCR and normalized to *Gapdh* levels. **(B)** U2OS cells were treated with 4mM hydroxyurea for 18 hr, and then released to resume growth for the indicated times. RNA was extracted, and *Gasl1* RNA levels determined by Real-time RT-PCR, and normalized to *Gapdh* levels.

Having established that activation of ectopic E2F1 induces expression of GASL1, we examined whether endogenous E2Fs are capable of influencing its expression. To this end, we took advantage of the Human Papilloma Virus oncoprotein E7, which disrupts RB/E2F complexes, resulting in activation of endogenous E2Fs. In line with our data based on ectopic E2F1, expression of E7 in WI38 human fibroblasts elevated the levels of GASL1, compared to cells expressing mutant E7, which does not disrupt RB/E2F complexes (Figure 2A).

E2F1 is a pivotal regulator of cell cycle progression and many of its target genes exhibit a cell cycle-regulated pattern of expression. Therefore, we next determined whether GASL1 is differentially expressed along the cell cycle. To that end, we examined its RNA levels in U2OS cells that were synchronized using hydroxyurea (HU), which inhibits ribonucleotide reductase thereby synchronizing cells in the G1/S transition. The cells were then released from the hydroxyurea block, and their re-entry into the cell cycle was monitored (Supplementary Figure 3). Hydroxyurea treated cells were arrested in late G1, and this resulted in a 4 fold increase in GASL1 RNA levels, compared to unsynchronized cells. Moreover, as the cells were released from the hydroxyurea block, GASL1 RNA levels further increased 4 hours after the release, as cells entered S phase, followed by a gradual decline (Figure 2B and Supplementary Figure 3).

**Figure 3:**
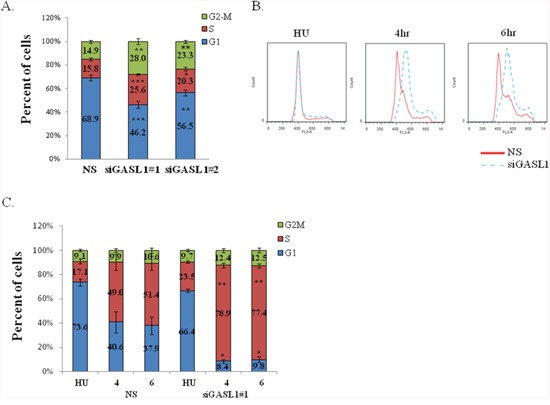
Silencing of *GASL1* enhances G1 exit **(A)** U2OS cells were transfected with non specific siRNA (NS) or two distinct *GASL1*-specific siRNAs (si*GASL1*#1 or #2) and cells were analyzed by FACS 72h post-transfection. Percentages of cells in G1, S, and G2/M cell-cycle phases are depicted. Results shown are an average of six independent experiments (*p<0.05,**p<0.01, ***p<0.005; two-tailed Student's t-test). **(B)** U2OS cells were transfected with either a nonspecific siRNA (NS) or siRNA directed against *GASL1*. After 48 hr, cells were left untreated or incubated with Hydroxyurea (4mM, 18hr). The cells were then allowed to resume growth for 4 or 6 hours in fresh media. Cells were analyzed by FACS using Propidium-Iodide (PI) staining. **(C)** The bar graphs depict the average cell cycle distribution from four independent experiments in which cells were treated as in C (*p< 0.05, **p<0.01; two-tailed Student's t-test).

Many E2F-regulated genes encode proteins that affect cell cycle progression [23], and a number of E2F-regulated non-coding RNAs were also shown to regulate the cell cycle [21, 22, 24]. Therefore, we next tested whether GASL1 plays a role in this biological process. Indeed, reducing the endogenous GASL1 RNA levels in U2OS cells using two distinct siRNAs reproducibly resulted in cell cycle redistribution (Figure 3). Specifically, knockdown of GASL1 (Supplementary Figure 4A) resulted in a significant decrease in the number of cells in the G1 phase of the cell cycle and a concomitant increase in the number of cells in the other phases of the cell cycle, suggesting that GASL1 may negatively regulate G1 exit (Figure 3A). Similar results were also observed in HeLa cells (Supplementary Figure 4B, 4C). To directly determine whether GASL1 plays a role in G1-phase exit, U2OS cells were synchronized with hydroxyurea (HU) and then released from the hydroxyurea block, and their re-entry into the cell cycle was monitored. Importantly, as GASL1-silenced cells were released from the hydroxyurea block, many more cells exited G1 and transitioned into S phase (Figure 3B, 3C). These results support our earlier observations, and indicate that silencing GASL1 enhances G1 exit and cell cycle progression.

**Figure 4:**
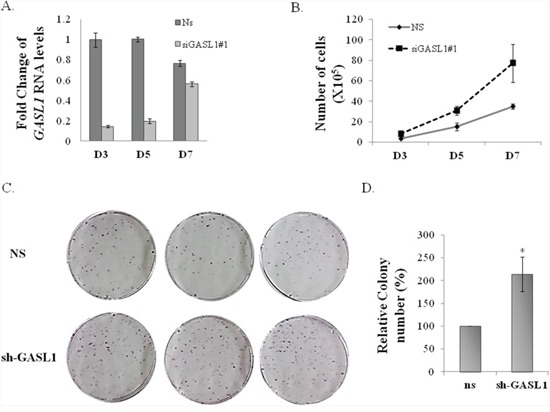
Silencing of GASL1 enhances cell proliferation **(A)** U2OS cells were transfected with siRNA directed against *GASL1* or a non specific siRNA (NS). Total RNA was extracted from the cells and the level of *Gasl1* RNA was determined by real time qPCR, and normalized to *Gapdh*. **(B)** U2OS cells were transfected with either a non specific siRNA (NS) or *GASL1*-specific siRNA and cells were counted at the indicated times post transfection. The cell numbers shown are an average of three independent experiments, each performed in triplicates. **(C)** U2OS cells stably expressing shRNA against *GASL1* or a non specific shRNA were seeded at 5000 cells/dish, and cultured for 3 weeks. Then, the plates were Giemsa stained and the number of colonies determined. The assay was performed in triplicates. **(D)** The average numbers of colonies of four independent experiments performed as described in **(C)** (*p<0.05. two-tailed Student's t-test)

Next, we wished to further examine GASL1 function and test the effect of its prolonged silencing on cell proliferation and viability. To this end, we employed cell counting as well as colony assay, using U2OS cells in which GASL1 was silenced. As can be seen in Figure 4A significant silencing of GASL1 persisted for at least 5 days after transient transfection of the siRNA, however with weaker efficiency over time. This prolonged silencing of GASL1 enhanced U2OS cell growth by more than 100% over 7 days (Figure 4B). Similar results were obtained using the MTT assay (Supplementary Figure 5A). In addition, prolonged silencing of GASL1 using U2OS cells stably expressing an shRNA resulted in a 2-fold increase in the number of colonies as determined by a colony assay (Figure 4C, 4D and Supplementary Figure 5B). These results are in agreement with the effect of GASL1 silencing on cell cycle distribution, as demonstrated earlier (Figure 3A).

**Figure 5:**
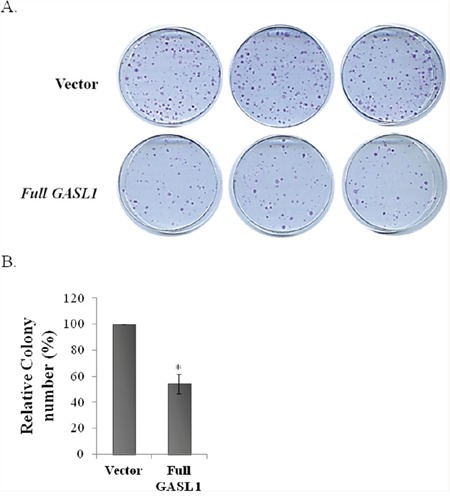
Ectopic expression of *GASL1* inhibits colony formation **(A)** U2OS cells (3×10^5^) stably expressing *GASL1* or empty vector were cultured for 2 weeks. Then the plates were Giemsa stained and the number of colonies determined. The assay was performed in triplicate. **(B)** The average numbers of colonies from three independent experiments performed as described in A (*p<0.05. two-tailed Student's t-test)

To further analyze the biological function of GASL1, full length GASL1 was cloned and the effects of its ectopic expression on cell growth were studied. The 3′-cDNA end and 5′-cDNA end of GASL1 were determined by RACE to be chr8:103,819,901 and chr8:103,823,335, respectively (data not shown), and GASL1 was shown to be 1536 bases long. Ectopic expression of GASL1 led to a significant and reproducible decrease in the number of colonies observed in a colony assay (Figure 5A, 5B). These data are in agreement with the enhancement of colony formation following GASL1 silencing (Figure 4C, 4D). Furthermore, these data indicate that the ectopically expressed GASL1 does not have to be transcribed at the chromosomal location of the endogenous lncRNA in order to exert its activity. This is in agreement with our cell fractionation experiments, which indicated that endogenous GASL1 is present mainly in the cytoplasm (Supplementary Figure 6)

As mention previously, our results suggest that E2F1 and GASL1 may function in a negative feedback loop, in which E2F1 activates expression of GASL1, and GASL1 inhibits G1/S transition and cell proliferation, two well-established biological functions of E2F1.

**Figure 6:**
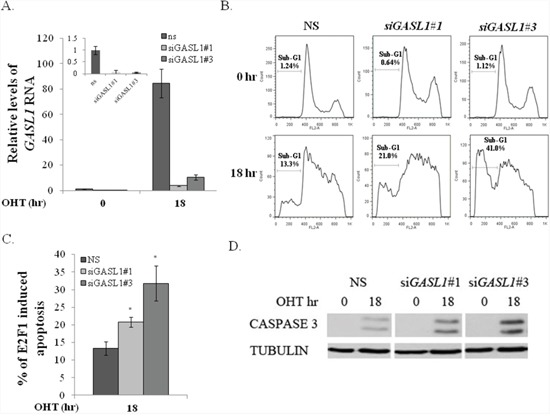
*GASL1* silencing promotes E2F1-induced apoptosis **(A–D)**. U2OS cells expressing ER-E2F1 were transfected with two distinct *GASL1*-specific siRNAs (si*GASL1*#1 or #3) or a non specific siRNA (NS) for 48 hours. After 24 hr, cells were treated with or without OHT (100nM) for 18hr. **(A)** Total RNA was extracted from the cells, and the level of *Gasl1* RNA was determined by real time qPCR and normalized to *Gapdh*. Inset - *Gasl1* RNA levels in cells that were not treated with OHT. **(B)** FACS analysis. Percentage of cells with Sub-G1 DNA content is indicated. **(C)** Average percentage of E2F1-induced apoptosis determined by percentage of cells with sub-G1 DNA content. Graph shows the average of three independent experiments (*p< 0.05. two-tailed Student's t-test). **(D)** Western blot monitoring cleaved CASPASE 3.

To further study the role of GASL1 in E2F1-mediated biological processes, we reduced endogenous GASL1 RNA levels in U2OS cells expressing ER-E2F1 using two distinct siRNA, and examined the effect on E2F1-induced apoptosis. Introduction of each of these two siRNAs resulted in significantly reduced basal levels of GASL1 RNA and inhibited its up regulation by E2F1 compared to non-specific siRNA (Figure 6A). In line with previous reports, activation of ectopic E2F1 resulted in apoptosis, which was evaluated by monitoring the percentage of cells with sub-G1 DNA content, and also by monitoring the levels of cleaved caspase 3 (Figure 6B, 6C, 6D). The knockdown of GASL1 significantly enhanced E2F1-induced apoptosis, as evident by the reproducible increase in the percentage of cells with sub G1 DNA content, as well as the increase in cleaved caspase 3 (Figure 6B, 6C, 6D). Silencing of GASL1 specifically affected E2F1-induced apoptosis as it did not have a significant effect on apoptosis induced by other stimuli such as Cisplatin or Etoposide (data not shown). These data support the notion of a negative feedback loop between E2F1 and GASL1.

**Figure 7:**
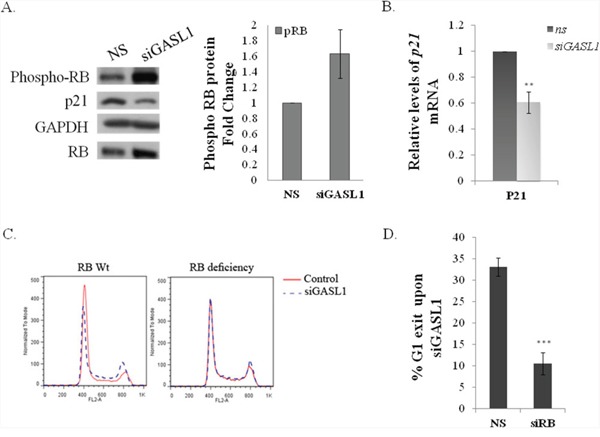
The effect of GASL1 silencing on cell cycle distribution requires pRB **(A)** U2OS cells were transfected with siRNA directed against *GASL1* or a non specific siRNA (NS) for 48 hours. Proteins were extracted from the cells and the levels of RB, phosphorylated RB and p21 were monitored by western blot analysis. The graph depicts fold change in phosphorylated RB protein level in the *GASL1* depleted cells. **(B)** U2OS cells were transfected with siRNA directed against *GASL1* or a non specific siRNA (NS) for 48 hours. RNA was extracted from the cells. The levels of *p21* mRNA were determined by real time qPCR and normalized to *Gapdh*. The average of four independent experiments is presented (*p<0.05,**p<0.01; two-tailed Student's t-test). **(C–D)** U2OS cells were transfected with siRNA directed against *GASL1* (si*GASL1*) or/and against RB (siRB) or a non specific siRNA (NS) for 48 hours. **(C)** FACS analysis **(D)** Percentage of G1 exit, determined by comparing the percentage of cells in G1 with and without silencing of GASL1. Results shown are an average of four independent experiments is presented. (***p< 0.005; two-tailed Student's t-test).

In a search for the molecular mechanism(s) underlying the effect(s) of GASL1 on E2F activity, we examined whether it regulates the RB/E2F pathway. To test this hypothesis we examined the effect of GASL1 knockdown on the RNA and protein levels of pivotal players in the RB/E2F pathway. mRNA and protein levels of E2F1 itself were not significantly affected by silencing GASL1 (data not shown). However, while silencing of GASL1 did not have a significant effect on RB mRNA levels (Supplementary Figure 7A), it resulted in a significant increase in the levels of phosphorylated RB (Figure 7A). Also, silencing of GASL1 led to a reduction in the mRNA and protein levels of p21, a pivotal CKI (Figure 7A, 7B and Supplementary Figure 7B). The reduction in p21 levels and in RB phosphorylation are most probably not merely a reflection of changes in cell cycle distribution, as they were also detected in GASL1 silenced cells that were arrested by Hydroxyurea (Supplementary Figure 7C and data not shown). These data suggest that GASL1 depletion affects cell cycle distribution via pRB. In agreement with this notion, silencing of Rb in U2OS cells significantly inhibited the effect of GASL1 silencing on cell cycle distribution and in particular on G1 exit (Figure 7C, 7D). Furthermore, silencing of GASL1 in SAOS-2 cells, which lack functional pRB, did not affect cell cycle distribution (Supplementary Figure 7E). Altogether, these data support the involvement of GASL1 in a negative feedback loop in the E2F/RB pathway. The molecular nature of this activity of GASL1 remains to be determined.

**Figure 8:**
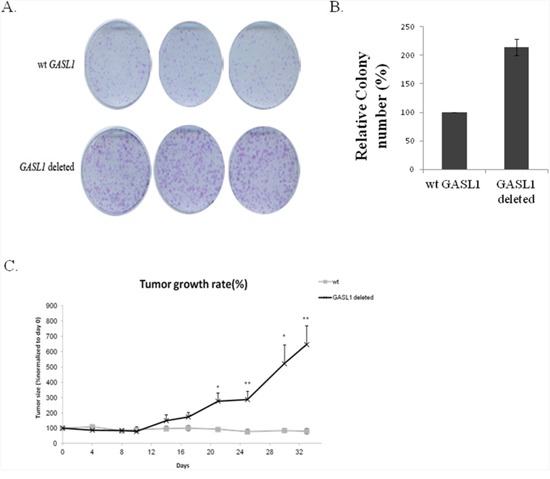
GASL1 deletion enhances colony formation as well as tumor growth in a xenograft mouse model **(A)** Colony formation assay of GASL1 deleted U2OS cells. After 2 weeks, cells in each well were fixed and counted. **(B)** The average numbers of colonies of two independent experiments performed as described in **(A)**. **(C)** Mice were injected with either WT U2OS or GASL1 deleted U2OS cells, six mice in each group. Growth curve drawn by measuring tumor volumes on the indicated days. (Error bars represent the SEM, *p < 0.05, **p<0.01 two-tailed Student's t-test).

Next, to confirm that the expression level of GASL1 affects tumor growth *in vivo*, we generated U2OS cells deleted for GASL1 using CRISPR/Cas9. Colony formation assays with these cells showed, as expected, that deletion of GASL1 promoted colony formation (Figure 8A, 8B). Then, U2OS cells and GASL1-deleted U2OS cells were injected subcutaneously to NSG mice. Monitoring of tumor size clearly demonstrated that tumors from GASL1-deleted cells were significantly larger in size and volume compared with control tumors (Figure 8C). These results demonstrate that GASL1 plays a crucial role in osteosarcoma cell growth *in vivo*.

**Figure 9:**
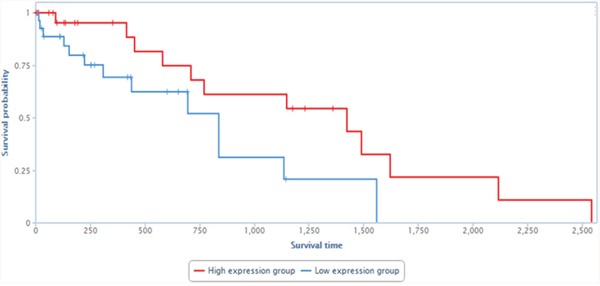
Low levels of *GASL1* are associated with poor prognosis in liver cancer patients Data derived from 200 liver cancer samples was stratified into two groups according to the RNA levels of GASL1. *GASL1* expression was based on reads within chr8:103,819,901-103,823,335 and the presented analysis was performed using TANRIC [25]. P<0.04

Finally, to evaluate the physiological relevance of GASL1 to human tumors, we tested whether its levels correlate with patient survival in a cohort of 200 hepatocellular carcinoma patients [25]. Remarkably, upon stratification of this cohort into two subgroups: one with high levels of GASL1 and the other with low levels of GASL1, it became apparent that the group with high levels of GASL1 exhibited significantly increased survival (Figure 9). These data are consistent with the findings that high GASL1 levels lead to inhibition of cell growth (Figure 5).

## DISCUSSION

lncRNAs are emerging as pivotal regulators of many biological processes, and a number of lncRNAs were shown to regulate the cell cycle [5]. We identify here a novel lncRNA that affects cell cycle progression, which we named GASL1 for Growth-arrest Associated lncRNA 1. We show here that expression of GASL1 is upregulated by E2F1, and its silencing enhances G1/S transition, cell proliferation and tumor growth *in vivo*, while its ectopic expression inhibits cell proliferation. Silencing of GASL1 also results in elevated levels of phosphorylated pRB and enhancement of E2F1-induced biological activities. Moreover, the ability of GASL1 silencing to effect cell cycle distribution requires functional pRB. Taken together, these data suggest an E2F/GASL1 negative feedback loop.

### The E2F1-regulated GASL1 is a modulator of cell cycle progression

E2Fs are transcription factors best known for their involvement in the timely regulation of protein-coding genes required for cell cycle progression [23]. Though E2F1 is particularly known as a positive mediator of the G1/S transition, a number of pivotal negative regulators of cell cycle progression are transcriptionally activated by E2Fs. These include p18^INK4c^ [26], p19^INK4d^ [27], and pRB [28]. Recent studies indicate that E2Fs also regulate the expression of non-coding RNAs, including microRNAs and lncRNAs that control cell cycle progression [21, 29, 30]. Thus far, six lncRNAs were shown to exhibit E2F-regulated expression. These include H19, an lncRNA encoded by an imprinted gene whose levels are elevated in many human cancers [19]; ANRIL, which is located at the tumor suppressor locus encoding p16^INK4A^ and p15^INK4B^, and represses the expression of these two tumor suppressors [21, 31, 32]; ERIC, which was shown to regulate apoptosis [20]; the anti-sense Khps1 that positively regulates the expression of the oncogene Sphk1 [33]; MA-linc1, which plays a role in M/G1 transition and enhances apoptotic cell death induced by the antimitotic drug, Paclitaxel [22]; and LINC00668 which, similarly to ANRIL, promotes cell proliferation through epigenetic silencing of CKIs [34]. GASL1 now joins this short list of E2F-regulated lncRNAs, and to our knowledge, this is the first example of an E2F-regulated lncRNA that inhibits cell cycle progression and cell proliferation.

Of note, ectopic expression of GASL1 led to inhibition of cell growth as assayed in our colony assay, which is the opposite effect of the knock down that enhanced colony formation. This result further validates our conclusion that GASL1 inhibits cell proliferation.

### A GASL1/E2F1 negative feedback loop

Our data suggest that GASL1 and E2F1 form a negative feedback loop in which E2F1 positively regulates GASL1 expression while GASL1 negatively regulates E2F1-mediated activities, including both G1/S transition and E2F1-induced apoptosis. The molecular mechanism(s) underlying the effect of GASL1 on the RB/E2F pathway await further studies. However, our data demonstrate that silencing of GASL1 elevates the levels of phosphorylated pRB, suggesting that GASL1 negatively regulates pRB phosphorylation thereby inhibiting E2F activity. The importance of pRB in mediating the activity of GASL1 is further supported by our observation that co-silencing of Rb inhibits the effects of GASL1 silencing on G1/S transition. Furthermore, in SAOS-2 cells that lack functional pRB the knockdown of GASL1 did not affect cell cycle distribution and G1/S transition. The exact mechanism by which GASL1 affects pRB phosphorylation remains unknown at this time.

Of note, a negative feedback loop involving a lncRNA and a transcription factor was reported in the case of the lncRNA RoR, which is transcriptionally regulated by p53 and is a strong negative regulator of p53 [35].

The RB/E2F pathway is highly regulated and includes a number of negative feed back loops. For example, the RNA binding protein RBM38, as well as the microRNAs miR-15, miR-16, miR-449a and miR-449b are all positively regulated by E2F1 and restrict E2F1-induced cell cycle progression [24, 29, 36, 37]. Also, the lncRNA, ERIC, which was shown to be upregulated by E2F1, inhibits E2F1-induced apoptosis [20]. Our data indicate that the lncRNA GASL1 is a component of a novel negative feedback loop in the RB/E2F pathway.

In agreement with the inhibitory function of GASL1 on cell cycle progression and cell growth that we observed both in tissue-culture cells and in xenografts, analysis of liver cancer patients suggests that GASL1 is a relevant player in human cancer. Specifically, high levels of GASL1 are associated with increased survival.

Taken together, we show here that the lncRNA GASL1 is regulated by E2F1 and can restrain E2F1 activity. We propose that this lncRNA serves to fine-tune E2F1 activity, thereby preventing excessive E2F1 activity that may harm cells and result in uncontrolled proliferation.

## MATERIALS AND METHODS

### Cell lines and culture

U2OS and SAOS-2 human osteosarcoma cells were grown in Dulbecco's modified Eagle's medium supplemented with 5% or 10%, respectively, fetal bovine serum (FBS). WI38 human embryonic lung fibroblasts and HeLa cervical cancer cells were grown in minimal essential medium supplemented with 10% fetal bovine serum, 2 mM L-glutamine, 1 mM sodium pyruvate and non-essential amino acids. H1299 human lung adenocarcinoma cells were grown in RPMI 1640 medium supplemented with 5% fetal bovine serum. Cells were maintained at 37°C in a humidified atmosphere containing 8% CO_2_. To induce activation of ER-E2F1 or ER-E2F1-E132, cells were treated with 100 nM 4-hydroxytamoxifen (4-OHT, Sigma) for the times indicated. Hydroxyurea (Sigma) was used at 4mM for 18 hours. Where indicated, cycloheximide (Sigma) was used for 8hr at 10μg/ml.

### Quantitative PCR (Real-Time qPCR)

Total RNA was extracted from the cells using the Tri Reagent method. Real-time quantitative PCR (qPCR) was done using SYBR Green PCR Master Mix (Quanta) and the following primer pairs: Gapdh: 5′-CATGTTCCAATATGATTCCACC-3′ and 5′-GATGG GATTTCCATTGATGAC-3′ Gasl1: 5′-CTGAGGCCA AAGTTTCCAAC-3′ and 5′-CAGCCTGACTTTCCCT CTTCT-3′ p21: 5′-TCACTGTCTTGTACCCTTGTGC-3′ and 5′-GGATTAGGGCTTCCTCTTGG-3′ Rb: 5′-GAA GCAACCCTCCTAAACCAC-3′ and 5′-TCATTTCTGC CAGTTTCTGCT-3′ Malat1: 5′-TGGGGGAGTTTCG TACTGAG-3′ and 5′- TCTCCAGGACTTGGCAGTCT-3′ Tubulin: 5′-GGAGCTGATGGAGTCAGTGATG-3′ and 5′-CAGCTCTCAGCCTCCTTTCTG-3′ All real-time reverse transcriptase PCR (RT-PCR) reactions were performed using the Applied Biosystems StepOnePlus Real-Time PCR Systems Machine. Results are presented as mean and SD for duplicate runs. Relative transcript levels were determined by normalization to Gapdh mRNA.

### Western blotting

Cells were lysed in lysis buffer [50 mmol/L Tris (pH 7.5), 150 mM NaCl, 1 mM EDTA, 1% NP40] in the presence of protease inhibitor cocktail (Roche) and phosphatase inhibitor cocktails I and II (Sigma). Equal amounts of protein, as determined by the Bradford assay, were resolved by electrophoresis through an SDS 10% or 12.5% polyacrylamide gel and then transferred to a PVDF membrane (Millipore). The membrane was incubated with one of the following primary antibodies: anti-cleaved CASPASE-3 (#9664S, Cell Signaling); anti-TUBULIN (T9026, Sigma); anti-GAPDH (sc-25778, Santa Cruz Biotechnology); anti-phospho-RB (#9308, Cell signaling); [5]-anti-p21 (sc-397, Santa Cruz Biotechnology). Binding of the primary antibody was detected using an enhanced chemilluminescence kit (ECL Amersham).

### Plasmids

The plasmids pBabe-neo-HA-ER-E2F1, pBabe-puro-HA-ERE2F1-E132 [38, 39], pBABE-puro-16E7, pBABE-puro-E7-dl21-35, prcCMV-HA-E2F1(E132), were used.

An shRNA directed against GASL1 (5′- GACGTGTCAGGACCTTCGT) was cloned into the retroviral vector, pRETRO-SUPER [40]. To express GASL1 a 1536 bp fragment of GASL1 (based on RACE analysis) was cloned into pEFIRES-P vector [41]. For CRISPR/Cas9-mediated deletion pSpCas9(BB)-2A-GFP was used.

### Transfection and infection procedures

To generate Retroviruses, cells (2 × 10^6^) of the 293T packaging cell line were cotransfected with ecotropic packaging plasmid pSV-EMLV (2 μg), which provides packaging helper function, and the relevant plasmid (2 μg). The transfection was done using Poly Jet transfection reagent according to the manufacturer's instructions (Signagen). After 6hr, the transfection medium was replaced with fresh Dulbecco's modified Eagle's medium supplemented with 5% fetal bovine serum. Cell supernatants containing retroviruses were then collected. For infection, cells were incubated for 5hr at 37°C in 4.5 mL of retroviral supernatant supplemented with polybrene (8μg/mL, Sigma H9268). Then, 5.5 mL of medium was added, and after a further 24hr, the medium was replaced with fresh medium containing puromycin (2μg/mL, MEGAFARM P-1033-SOL).

When transfecting U20S cells with siRNA, Interferin transfection reagent (PolyPlus-transfection) was employed according to the manufacturer's instructions. The siRNAs were synthesized by Sigma-Aldrich: siGASL1#1 GCUGCAAGGGAAAUGACAUCGGUUA, siGASL1#2-CGUGGGACUACUAGAAGGAAAGAUA, siGASL1#3 -CCUGAGGCUAGAGGGUCUAAGAGAA, siRB1 CC UAGUUCACCCUUACGGA and a control sequence (siRNA universal negative control #1). Experiments were performed 48 or 72 hours after transfection with siRNAs as indicated.

For transfecting U20S cells with plasmids, polyjet transfection reagent (Signagen) was employed according to the manufacturer's instructions.

### Extraction of nuclear and cytoplasmic RNA

RNA was extracted from the nucleus and cytoplasm according to the Invitrogen nuclear extraction protocol. Cells were washed twice with PBS, and resuspended in 500μl 1x Hypotonic Buffer. After 15 min incubation on ice, 10% NP40 detergent was added, and the samples were vortexed. Then, the homogenate was centrifuged for 10 minutes at 3,000 rpm at 4°C. The RNA from the pellet, containing the nuclear fraction, was extracted by the Tri Reagent method. The RNA from the supernatant, containing the cytoplasmic fraction, was extracted by the Phenol-Chloroform method.

RNA levels of the nuclear and the cytoplasmic fractions were monitored by RT Real Time PCR and were normalized to levels of external DNA.

### Cell proliferation and viability assay (MTT)

U2OS cells transfected with nonspecific siRNA or siRNA against *GASL1* were grown for the indicated times and then stained for 40 min at 37°C with MTT (3-[4,5-dimethylthiozol-2-yl]-2,5diphenyltetrazoliumbromide); absorbance was measured at 570 nm using a TECAN spectrophotometer.

### Colony formation assay

U2OS cells stably expressing a non-specific shRNA or an shRNA against *GASL1* were seeded (5000 cells/plate) and cultured for 3 weeks to form colonies.

U2OS cells (3×10^5^) were seeded and transfected with empty vector or a *GASL1* expression vector. The cells were cultured with puromycin (2.5μg/μl, Megapharm) for 2 weeks to form colonies.

GASL1-deleted U2OS cells were seeded (1000 cells/plate) and cultured for 2 weeks to form colonies.

Colonies were fixed with 100% ethanol, and stained with 10% Giemsa for 15 minutes.

### Cell cycle distribution assay

#### Propidium iodide (PI) staining

Trypsinized cells were fixed with 70% ethanol at 4°C overnight, washed with PBS, and stained with 5 mg/ml propidium iodide (Sigma) in the presence of 50 μg/ml RNase A (Roche). After incubation for 15 min at room temperature, fluorescence was measured using a Becton Dickinson flow cytometer.

### Rapid amplification of cDNA ends (RACE)

5′ and 3′ RACE were performed using the 5′/3′ RACE kit (Roche). 1 μg RNA from U2OS cells was used for the 5′ and 3′ RACE. A single oligo d(T)- primer and anchor primer were used for both RACE analyses. For 5′ RACE analysis, the anchor primer was 5′-CAGACCAATAAAATCAGATTTCC; for 3′ RACE analysis, the anchor primer was 5′- TCACAAGCTTCCTGATGCG. In addition, one nested lower primer, SP1short: 5′- CAAATTCTTC ACCTGGTCTCG, was used for 3′ RACE.

CRISPR/Cas9-mediated deletion

Generation of GASL1–deleted U2OS Cells. Two sets of small guide RNA#1-GGCAACCTACTGCGCACCAG, sgRNA#2-GCCAAAGTTTCCAACGGGAA were created using the CRIPSR Design Tool (http://tools.genome-engineering.org). Each sgRNA was cloned into pSpCas9 plasmid. Deletions were created by using two gRNAs that direct Cas9 to cleave out the specific region of DNA. The two plasmids were transfected into U2OS cells with PolyJet (Signagen). To validate the deletion, genomic DNA was isolated from the cells 2 weeks post transfection, with a QuickExtract kit from Epicenter. The deletion was confirmed by sequencing. Primers (for sequencing): 5′CAAACCGAGAAGCAGGAAAG,5′ CATGCAGTTCTCAACCCACA.

### Animal model and injections

NSG mice aged 8 weeks were subcutaneously injected with 2.5×10^6^ GASL1 knock-out U2OS or Wild-Type U2OS cells using Cultrex® Basement Membrane Extract (3432-005-01, Trevigen, USA), in a ratio of 1:1. The mice were observed over 56 days for tumor formation. Tumor growth was measured in a blinded fashion using a caliper and calculated using the following formula: [D X d2] X Π/6, where D is the largest tumor diameter and d its perpendicular one. All of the procedures were performed according to the guidelines of the university committee for animal welfare.

## SUPPLEMENTARY FIGURES


