# Supplementary material for: A novel lncRNA, GASL1, inhibits cell proliferation and restricts E2F1 activity
Source: Oncotarget. 2017 Mar 3;8(14):23775–86. doi: 10.18632/oncotarget.15864 (PMC5410343; doi:10.18632/oncotarget.15864)
Supplement: Supplementary file 1 [file oncotarget-08-23775-s001.pdf]

A novel lncRNA, GASL1, inhibits cell proliferation and restricts E2F1 activity

SUPPLEMENTARY FIGURES

```
GASL1 seq
TGCCGCAGGAGGGCGCTGTGGAGTACAGGCACATGCAGAAGGTGTGGCGAGGGCTTCCTGGTGGAGCTGGCAGTAGGGCAGGGA
CCTTGAAAAGGAATGATTTGAACAGATCGGAAAAGACATTCCAGACAGAAACAAATGCCTGAAACCCAAACCGAGAAGCAGGAA
AGAGCAGACTGCGTTCTCGGAATGGCAGGGAGTCCAGTTTGACTGGAAGACCTGAGGCTCAGAGGCGCATCACGGCCCAGATGC
AGACCACAGAGGCAGGCACAGTGGCCCAGGAGGACCGAGGACCTGGAGTCACGGGGTTCCGCCCGCTGCCCCTGGGGAACCGAG
TCATGCCAGTTCCCAGACAGATGAATTCCTTCCAAGAATTCCACAGTCCTGCTCAATCCTAGCCGCCAAGCCTCCTGGCCTTGGAT
GGCTGCTGTCTAATGCAGATCCTCTTTCATCCAGATTTCAAATATTTTTCCTATTCTGTCCTTTGATCACTAACGTTTATGTTTATCT
ACATCAGCATCCCTCAATCCTGACGGCACTCTGAAATTATCTAGGGGTGCTTTAAAAAAGAAAAATGTTGATGCATGGCCCTCCTC
CAGGAAATCTGATTTTATTGGTCTGGGGTGGGGCCCAGGCATCAGCAGACTTTTAATGAGCAGTTAGAGAGGAGAACCACTGATG
GAGATGGTCACCAGGGAGTGTCCAGGTCAGACCGCCACAGAAGAGTGCAGCCTCCTGGCCTTCAGACCCAACTGGGACAAACCCT
GAGAACAGCCTTGGCAGACCTCCAGGGGCAGCTGGACTCCCTGAGGCTGGATGTTGCAGCCTCTCCCAGGCATCCACTCTGCAGC
TGAGGCCAAAGTTTCCAACGGGAAGGGAGGGTGAGGCTTCTATTTGTTTCCCAAGAGCCAGCTGACCTTTGAAGCTAGTGATAACCACCAC
AAACCTCACTGGTGGGAGAACGAGGCACTGCAGCCAGTTCTCATCAACCCCACATCCCTGCAGGGGGCCCCAAGGCGGATGTGTCACCATCCTC
TGCAACGCTGGGCCTCAGAGGTGGAGGAGGCCCAGCCATGGCCAGATGGGGATCTGGGGAACAGGTCCTCTGCTTTCTGGGAAAATAGAGGT
GATGATTCCTGGCCCCAAAGGTTCTTGTTGGGAGTCTTGAGATAAATCCCCTAGCAGTGCTCAGCATGGGGAGAATTTTCAGTGACTGTTCCCTT
CCTCCTGTTTTGCTAATAACTCTTTTCACAAATTTAGGAGAGTCTCCGAGTCTGGGCCTTGCCACTCCTCACTCAGCATCTGACCATCCCC
ACCTCCTGGATCTACAGGGTTCATAGAGGAGGACCCAGAAGAGGGAAAGTCAGGCTGAGGAACTTGGCCTCCCTGCTGCAAGGG
AAATGACATCGGTTACCAAGGAGGTGACCAGCTATCCTTTTCCAAGACTGAAAAGCAGAAATACGGCCCTGAGGCTAGAGGGTCT
AAGAGAACAATAGGAAGAATTTCCTGACAAAGGACAATGTTGCCTAAAGGACTCAGGAAGCTCTGTGAAAACAGGAGACTCCCG
AGCAGAGCACCAGACCCTTCTCCACCAGGCAGTGTTTTTCCCATATGACAGGTGAGAAAACTGAGGCTGAGAGACATGTAATAACTTGC
TGAAGCAGCAACCAGCACACCATATAGCTGAAATCTGTAGATCAGCAGTTAAAAGTGGTAGGGCAAGTCCTCTCTGACTTTCAGCGCTAAGAGC
CTAGGATTCTGTGTTTTTCTGCTTAAGCCTGGGAATCAGAGTCCTACAAAAGCCGCGCCTAATCCTGGGGGTGTGGGCTTTAAACCCAGAGAGAA
AGCATGTTATTGGAATACTCCTGCACTTTTCTCCCTTTGAATCCCACCACGGAAAGAAAATTCTGTGACGTGTACCCAGTATTTATTGGAGGGTAT
TTCCTAGCAAGAAAATGAGGGCGATTGACCAGGCACGGTGGCTCACGCCTGTAATCCCAGCACTTTGGGAGGTCAAGGTGAGTGGATCACATGA
GGTCAGGAGTTCGAGACCAGCCTGACCAACATGGAGAAACCCCGTCTCTACTAAAAATACAAAATTAGCCGGGCGTGGGGTGGCGCATGCCTGT
AATTCCAGCTACTCCTGAGGCTGTGGCAGCAGAATCGCTTGAACCCGGGAGACAGAGGTTGCAGTGAGTTGAGATCAAGCCATCGCACTCCAGC
CTGGGCAACAGAGTGAGACTCTGTCAAAAAAGAAAGTGTGGTACATCTATACTAATGGAGTACTATTTAGCCGTCAAAGAAAAAAAAGAAAAG
AAAAAGAAAGAGAAAGAAAGAAAGAAGGAAAGAAAGAAAGAAAAAGAAAATGAAGGCGATTGTCAACAGCCTCTGGAAATACAGAGCAGGG
TCCAGAAATGGCCCCTTCTTAGATGGCGGGCTGGACAGTGCTGCTCCTTCTTAGCCACTGGCGGCAGAAAGACAAAGCCTGGTAATTGTTCTTGG
GATTCTCCCTGTCACCTCTCCTTGTAGGTCACAAGCTTCCTGATGCGACAGCCCGTGGGACTACTAGAAGGAAAGATACTCCTTTTCCC
ATTTTACAGATCAGGAAACTGAGACTGGACAGAAACACCACCAGGATTGAACTGTGAATGGATCTGACTCCAACCCACAAATTCT
TCACCTGGTCTCGAAAAGAGGCCCTGGCAACCTACTGCGCACCAGAGGGGAACCCGCCCGCCGTCCCAGGCCGGGCTCTCCCTGCTGCG
GAGCGGCCCGGGCGCGGCGGGACAGGGAACGGAAGGAACGGGCTTGGCGCGCACTCGCCGAGGCCCAGGCAGGATTCGAGCGCCAGGGAGGC
GCCCATTTCCCTTCCCCCGCGCCTGGGAACGCGGTGCTGGGGGCGGGGCCCGACCGCTGCCCCCTCCAAACGCCTCGACCGCCAGGTGCCCCGAA
GCCCGGCCTCCCCTCCCACCCCCGCGCCTGTTCCCGGCGCAGCCCGAGCCGTGGCAGTCACGTCTCCCCATCCGGCCCCAAAGTAAGAGCCAGCC
GCAGCCGCGCCGACCCCGCGCTGGGTGGGAGGGCGCGGGGAGGGCGGGGAGGGCGCGGGGAGGGCGGGGCAAGGAGGTGAGGGGAGGGCGG
GTTGCCCGGGCCCCGCCCGCTGCGCCCAGGTCGGCCGGGGAGAGGCGCGGGCGAGTCGCAGCGCCGCAACCACCGCCCCTGCCGGGCGCTCGGC
GAGCGGGGGCAGGGCGGGGTGAGGCGTGGCCGGGGATGCCGCGAGGCCGCGCCTCCAGGGCTGTGCGCGGGCGGGTGTTGCTGGGGAGAAAA
GCGCGGCACGGCCCCGCCCGAGTCCTGCGGGCACAGCGCTCTCTGCTGGTGTGGGTTGAGAACTGCATGAGACTAATTAAAGACCA
CCATCCACTTAAAAAAAAAAAAAAA
```

Supplementary Figure 1: Sequence of *GASL1*. *GASL1* DNA sequence: chr8:103,819,901-103,823,335 (3435 bp), contains 4 exons. Gray- introns, Black- exons.

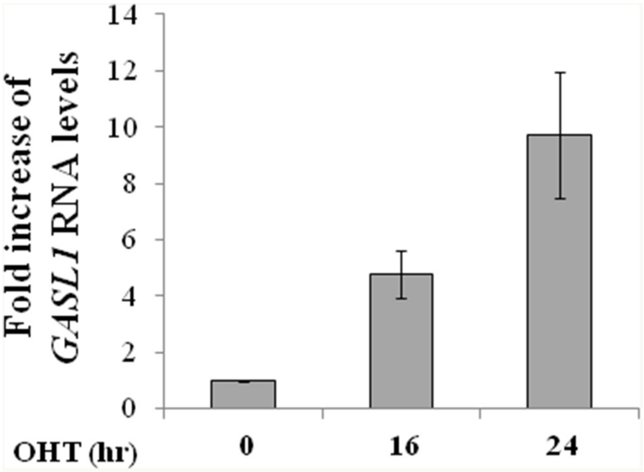

Supplementary Figure 2: Ectopic E2F1 upregulates GASL1 RNA levels. H1299 cells expressing ER- E2F1 (ER-E2F1) were left untreated or incubated with 4-OHT (100 nM) for the indicated times. RNA was extracted, and *Gasl1* RNA levels determined by Real-time RT-PCR and normalized to *Gapdh* levels. Graph shown presents the average of two independent experiments.

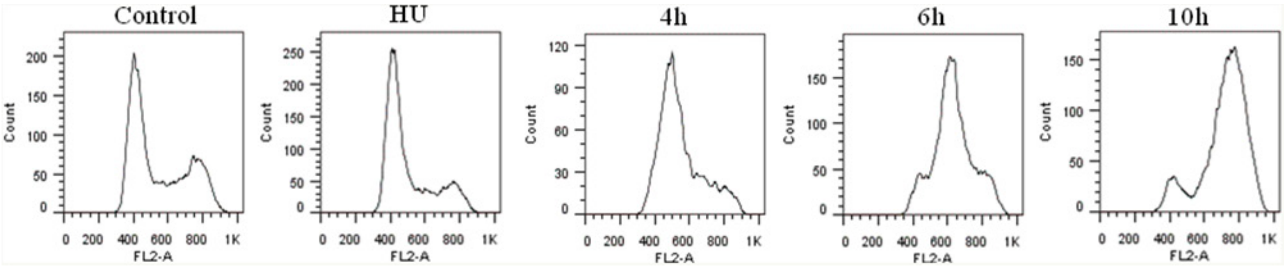

Supplementary Figure 3: Cell cycle distribution during and after a Hydroxyurea block. U2OS cells were treated with 4mM hydroxyurea for 18 hr. The cells were then allowed to resume growth for 4, 6 or 10 hours in fresh media. Cells were analyzed by FACS using Propidium-Iodide (PI) staining.

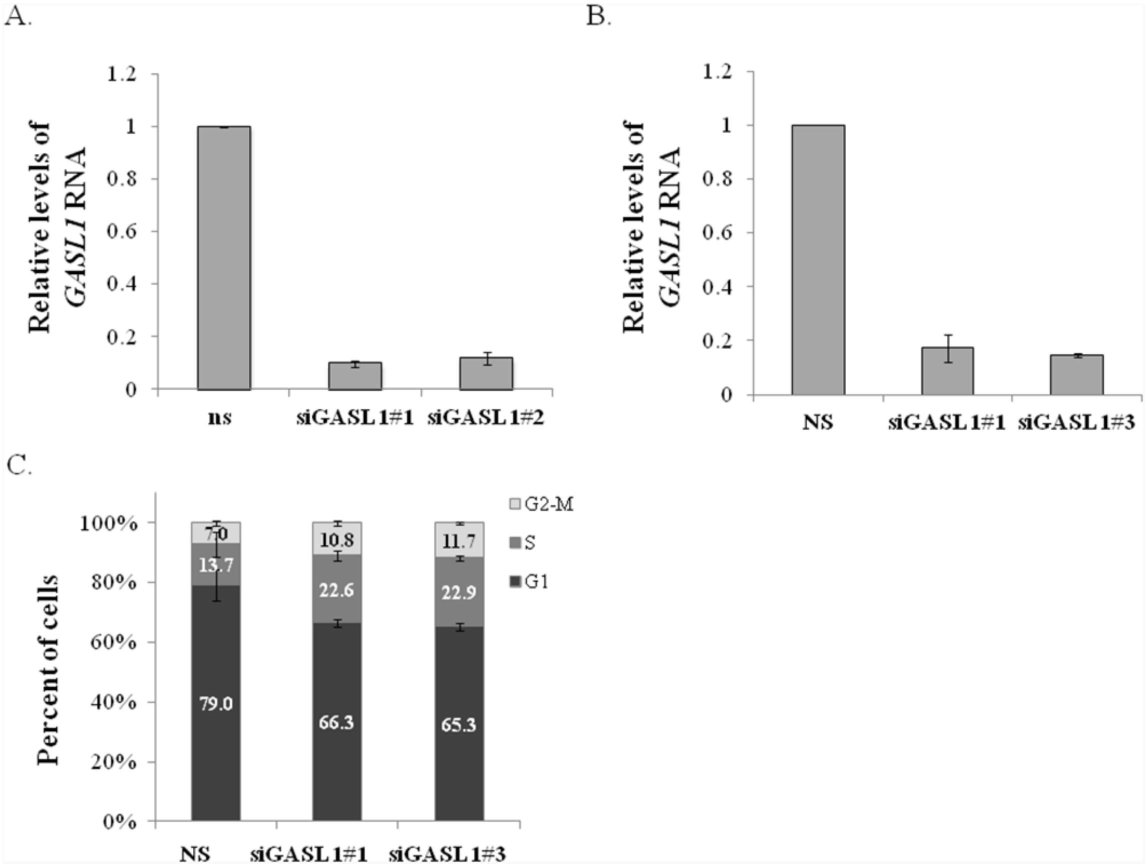

Supplementary Figure 4: siRNAs directed against GASL1 silence GASL1. (A) U2OS cells were transfected with non specific siRNA (NS) or two distinct *GASL1*-specific siRNAs (si*GASL1*#1 or #2) for 72h. RNA was extracted from the cells. The level of *Gasl1* RNA was determined by real time qPCR and normalized to *Gapdh*. The average of six independent experiments is presented. **(B)** HeLa cells were transfected with non specific siRNA (NS) or two independent *GASL1*-specific siRNAs (si*GASL1*#1 or #3) for 48h. RNA was extracted from the cells. The level of *Gasl1* RNA was determined by real time qPCR and normalized to *Gapdh*. The average of two independent experiments is presented. **(C)** HeLa cells were transfected with non specific siRNA (NS) or two distinct *GASL1*-specific siRNAs (si*GASL1*#1 or #3), and cells were analyzed by FACS 48h post-transfection. Percentages of cells in G1, S, and G2/M cell-cycle phases are depicted. The average of two independent experiments is presented.

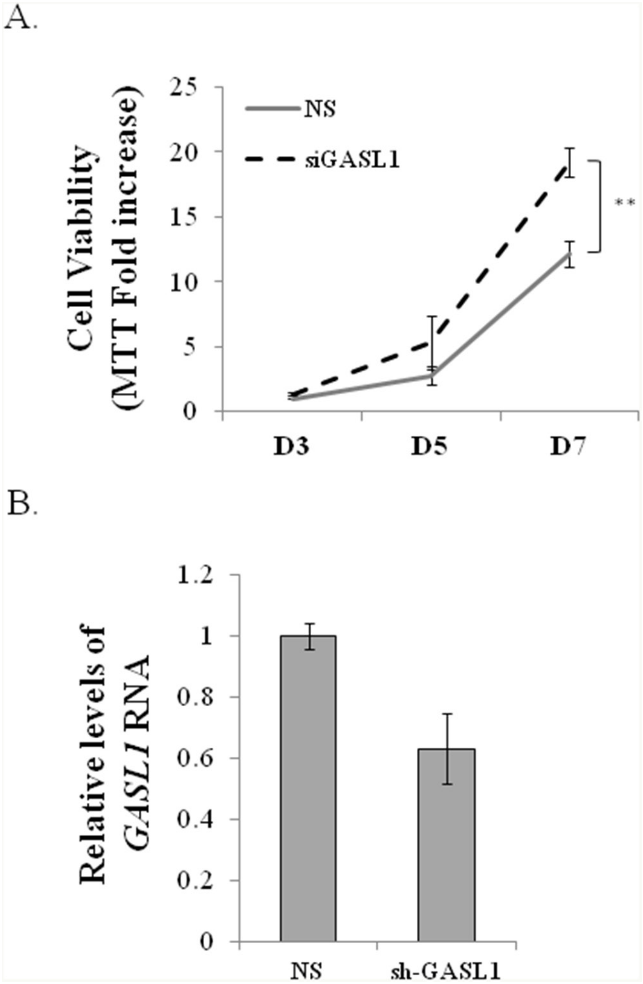

Supplementary Figure 5: Silencing of *GASL1* increases cell proliferation. **(A)** U2OS cells transfected with a non specific siRNA (NS) or *GASL1*-specific siRNA were grown for the indicated times (in days). Next, cell viability was determined by measurement of MTT absorbance at 570 nm. Data are presented relative to the absorbance of cells transfected with a non specific siRNA, 3 days after the transfection. This absorbance is set as 1. The average of three independent experiments is presented (**$p<0.01$; two-tailed Student's t-test) **(B)** RNA was extracted from U2OS cells stably expressing shRNA directed against *GASL1* or a non specific shRNA. The level of *Gasl1* RNA was determined by real time qPCR and normalized to *Gapdh*.

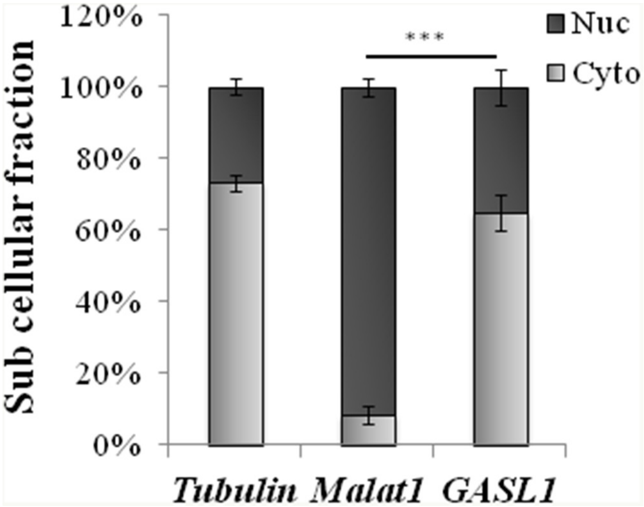

Supplementary Figure 6: *GASL1* subcellular localization. RNA was extracted from nuclear and cytoplasmic fractions of U2OS cells and the levels of nuclear control transcript (*Malat1*), cytoplasmic control transcript (*Tubulin*), and *Gasl1* were determined by Real-time PCR in nuclear and cytoplasmic fractions and normalized to levels of external RNA. The bar graph is presented as fraction of subcellular enrichment out of whole cell levels. The average of four independent experiments is presented (***p<0.005; two-tailed Student's t-test).

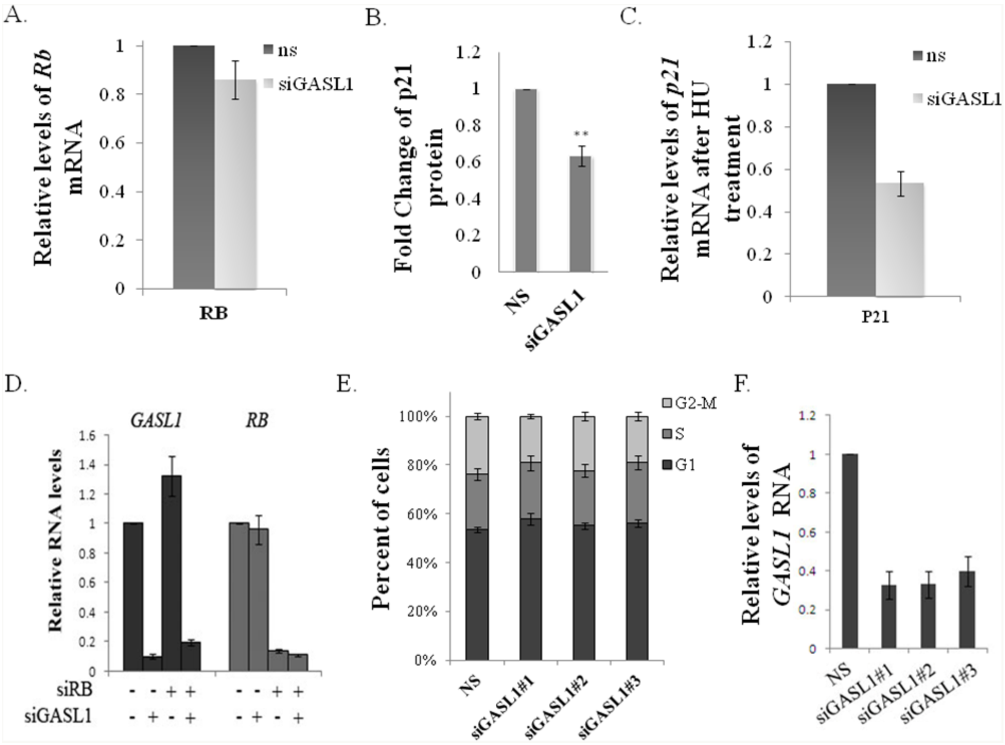

Supplementary Figure 7: Silencing *GASL1* reduces p21 levels and requires pRB to affect cell cycle distribution. (A) U2OS cells were transfected with an siRNA directed against *GASL1* or a non specific siRNA (NS). RNA was extracted from the cells after 48 hrs, and the level of *Rb* mRNA was determined by real time qPCR and normalized to *Gapdh*. The average of four independent experiments is presented. **(B)** U2OS cells were transfected with an siRNA directed against *GASL1* or a non specific siRNA (NS). After 48 hrs, proteins were extracted and western blot analysis was performed using antibodies directed against p21 and GAPDH. The graph depicts normalized protein levels of p21 quantitated using ImageJ. An average of four independent experiments is presented. (**$p<0.01$; two-tailed Student's t-test) **(C)** U2OS cells were transfected with either a nonspecific siRNA (NS) or an siRNA directed against *GASL1*. After 48 hrs, cells were incubated with Hydroxyurea (4mM) for 18 hours. RNA was extracted from the cells, and the levels of *p21* mRNA were determined by real time qPCR and normalized to *Gapdh*. Graph shows the average of two independent experiments. **(D)** U2OS cells were transfected with siRNA directed against *GASL1* (si*GASL1*) or/and against RB (siRB) or a non specific siRNA (NS) for 48 hours. RNA was extracted from the cells. The level of *Gasl1* and *Rb* RNA was determined by real time qPCR and normalized to *Gapdh*. The average of four independent experiments is presented. **(E-F)** SAOS-2 cells were transfected with non specific siRNA (NS) or three independent *GASL1*-specific siRNAs (si*GASL1*#1, #2 or #3) for 72h. **(E)** Cells were analyzed by FACS 72h post-transfection. Percentages of cells in G1, S, and G2/M cell-cycle phases are depicted. The average of three independent experiments is presented. **(F)** RNA was extracted from the cells. The level of *Gasl1* RNA was determined by real time qPCR and normalized to *Gapdh*. The average of three independent experiments is presented.
